# A Combinational Therapy for Preventing and Delaying the Onset of Alzheimer’s Disease: A Focus on Probiotic and Vitamin Co-Supplementation

**DOI:** 10.3390/antiox13020202

**Published:** 2024-02-05

**Authors:** Omme Fatema Sultana, Raksa Andalib Hia, P. Hemachandra Reddy

**Affiliations:** 1 Department of Internal Medicine, Texas Tech University Health Sciences Center, Lubbock, TX 79430, USA; osultana@ttuhsc.edu; 2 Nutritional Sciences Department, College of Human Sciences, Texas Tech University, Lubbock, TX 79409, USA; randalib@ttu.edu; 3 Public Health Department of Graduate School of Biomedical Sciences, Texas Tech University Health Sciences Center, Lubbock, TX 79430, USA; 4 Department of Speech, Language and Hearing Sciences, School Health Professions, Texas Tech University Health Sciences Center, Lubbock, TX 79430, USA

**Keywords:** probiotics, brain–gut axis, vitamins, neurodegenerative diseases, Alzheimer’s disease, cognitive impairment, synergistic effect, fortified food

## Abstract

Alzheimer’s disease is a progressive neurodegenerative disorder with a complex etiology, and effective interventions to prevent or delay its onset remain a global health challenge. In recent years, there has been growing interest in the potential role of probiotic and vitamin supplementation as complementary strategies for Alzheimer’s disease prevention. This review paper explores the current scientific literature on the use of probiotics and vitamins, particularly vitamin A, D, E, K, and B-complex vitamins, in the context of Alzheimer’s disease prevention and management. We delve into the mechanisms through which probiotics may modulate gut–brain interactions and neuroinflammation while vitamins play crucial roles in neuronal health and cognitive function. The paper also examines the collective impact of this combinational therapy on reducing the risk factors associated with Alzheimer’s disease, such as oxidative stress, inflammation, and gut dysbiosis. By providing a comprehensive overview of the existing evidence and potential mechanisms, this review aims to shed light on the promise of probiotic and vitamin co-supplementation as a multifaceted approach to combat Alzheimer’s disease, offering insights into possible avenues for future research and clinical application.

## 1. Introduction

The Alzheimer’s Association (2019) stated that Alzheimer’s disease (AD) dementia has a particular age-related onset and a progression of cognitive and functional decline that eventually results in mortality. AD accounts for up to 80% of dementia cases, making it the most prevalent cause of dementia by a significant margin [1]. While the overall mortality rate in the United States for heart disease and stroke is decreasing, there has been a notable increase in the proportion of deaths attributed to Alzheimer’s disease. Specifically, between 2000 and 2014, the percentage of deaths linked to AD has surged by 89% [2]. In 1906, Alois Alzheimer initially documented the condition in a report detailing the situation of Auguste Deter, a 51-year-old woman displaying symptoms such as cognitive decline, confusion, hallucinations, and various behavioral alterations [3]. Prominent characteristics of Alzheimer’s disease include notable impairments in memory, cognition, and motor skills. While the precise cause of Alzheimer’s disease remains elusive, some understanding of the molecular pathology has been gained. A notable pathological feature of AD involves the development of brain plaques composed of the amyloid-beta (Aβ) peptide [4]. These effects are primarily brought about by the accumulation of beta-amyloid plaques outside neurons and the presence of tau tangles within neurons. These processes can disrupt calcium balance, trigger neuroinflammation and oxidative stress, lead to vascular deterioration, and ultimately result in neuronal loss. AD is directly associated with the depletion of cells, malfunction of synaptic connections, and the formation of neuropil strands [5]. The amyloid cascade theory and the hypothesis of tau hyperphosphorylation are responsible for most of the newly suggested pathways in terms of the disease’s underlying causes. In 1991, John Hardy and David Allsop initially proposed the amyloid theory [6]. The amyloid precursor protein (APP), a vital element of the protein processing system, plays a significant role in the origin of Alzheimer’s disease. The primary contributors to the early onset of AD are mutations in proteins including presenilin 1 (PSEN1), presenilin 2 (PSEN2), and the APP. On the other hand, the late onset of AD results from a combination of factors, including lifestyle, aging, dietary choices, oxidative stress, pathological dysfunction, environmental influences, and the excessive expression of the apolipoprotein E4 (*ApoE4*) gene [7]. In Alzheimer’s disease, the accumulation of beta-amyloid is a common characteristic. Aβ is a peptide formed through the cleavage of the amyloid precursor protein carried out by a proteolytic enzyme. The APP is transported from the trans-Golgi network to the endosomal compartment through a process called clathrin-mediated endocytosis. During this process, a portion of the APP is returned to the cell surface within the endosome [8].

Additionally, the APP undergoes enzymatic hydrolysis by four different secretases, namely the α, β, η and secretases, in distinct ways to produce C-terminal fragments (CTFs). Under normal conditions, the first non-amyloidogenic pathway involves secretases. It generates products such as CTFs, the soluble ectodomain of the APP (sAPP), and other smaller fragments, which are neurotrophic and neuroprotective for nerve cells. The second pathway, which is known as the amyloidogenic pathway, entails the cleavage of the APP by a secretase to yield CTFs and the subsequent production of various lengths of A peptides by a secretase, including A42. A42 is more prone to aggregate and form plaques compared with A40 and is associated with more significant neurotoxicity [9,10]. An additional distinctive pathological feature of Alzheimer’s disease involves the emergence of neurofibrillary tangles in the brain, which are characterized by the presence of extensively phosphorylated tau proteins [11]. Tau has gained significant recent prominence, which is partially because several clinical trials of therapies targeting Aβ have not succeeded and also because tau pathology is more closely associated with cognitive impairments compared with Aβ lesions [12]. The microtubule-associated protein tau gene (*MAPT* gene) encodes the tau protein, which binds to the microtubules through an alternative [13]. Tau is integral in maintaining the synaptic integrity of the microtubule structure, thereby facilitating cytoplasmic transport and regulating neural signaling [14,15,16]. The development of tau pathology is an intricate and multifaceted process. In the brains of individuals with Alzheimer’s disease, tau undergoes hyperphosphorylation, altering the tubulin’s shape and impeding polymer formation, thus disrupting microtubules [17,18,19]. Interactions among tau proteins coupled with increased cytosolic tau levels result in the creation of insoluble straight filaments (SFs) and paired helical filaments (PHFs). Subsequently, these structures give rise to intraneuronal fibrillar deposits known as neurofibrillary tangles (NFTs) [17]. NFTs diminish synaptic connections, induce neurotoxicity, and disrupt cellular function [20,21]. In 2016, a comprehensive examination conducted as part of the Global Burden of Disease study revealed that approximately 43.8 million individuals across the globe were affected by Alzheimer’s disease [22]. Alzheimer’s disease affects an estimated 5 million older people in the United States, and this number is anticipated to increase significantly by 2050 [23]. Another research article reported that Asia currently accounts for 48% of global cases, and this percentage is expected to increase to 59% by the year 2050 [24]. Alzheimer’s disease will afflict more than 131 million individuals, thereby solidifying its position as a significant global health challenge in the years to come [24,25]. Dementia and AD are widely prevalent among elderly populations worldwide, and numerous studies have been carried out in both industrialized and non-industrialized nations to investigate this phenomenon. Within the United States, the occurrence of dementia is more elevated among African Americans and Latinos when compared with non-Latino white individuals [26]. Furthermore, World Alzheimer’s also reported in 2009 that there would be significantly sharper rises in the population of individuals affected by dementia in low- and middle-income nations as opposed to high-income countries. From 2015 to 2050, the number of people with dementia in what are currently considered high-income countries will grow by 116%. In contrast, there will be a 227% increase in upper-middle-income countries, a 223% increase in lower-middle-income countries, and a 264% increase in low-income countries during the same period [27]. Probiotics, when consumed in adequate quantities, are living bacteria that provide various health advantages. They function similarly to antibiotics, helping regulate the body’s pH balance, supporting the integrity of the intestinal lining, and enhancing the production of brain-derived neurotrophic factor (BDNF). BDNF, which is a vital protein, supports the survival of existing neurons and facilitates the growth and differentiation of new neurons, thereby contributing significantly to normal neurological development [28]. The communication between the gut microbiota and the brain is stimulated by the interconnectedness of the neurological system and the substances that can traverse the blood–brain barrier. One such example is the vagus nerve, which serves as a link connecting neurons in the brain with nerve cells in the intestine [29]. The interaction between CD8+ T cells and various peripheral immune cells, including B cells, natural killer T cells, and plasmacytoid dendritic cells, is influenced by the gut microbiome. CD8+ T cells play a training role for these immune cells. While gut-associated B cells are found in Peyer’s patches, the synthesis of mucosal IgA is significantly driven by the gut microbiota. Secretory IgA (SIgA) coats commensal bacteria and soluble antigens, thereby preventing their attachment to the host epithelium and infiltration into the lamina propria. This function establishes SIgA as a crucial intestinal barrier contributing to the maintenance of a mutually beneficial relationship between the host and the microbiota [30]. Historical shifts in the microbiome’s composition have indicated an elevated risk of Alzheimer’s disease. For example, the coevolution of humans with symbiotic microorganisms and recent shifts in the human environment and lifestyle, such as the advent of agriculture and industrialization, have brought about changes in the composition of symbiotic microbiota. These alterations encompass a significant reduction or loss of specific microorganisms, the introduction of new pathogenic microorganisms, shifts in relative abundances, and an overall decline in microbial diversity. These changes are implicated in the etiology of chronic inflammatory diseases. These things considered, there may be an increased risk of microbial translocation to the brain, progressively heightening the risk of AD immunopathology [31]. Contemporary research into the origins of Alzheimer’s suggests that imbalances in intestinal microbiota throughout one’s life may initiate a widespread inflammatory reaction and impact the immune response of microglia in the brain [32]. Recent research highlights the active involvement of gut bacteria in influencing various aspects of central nervous system (CNS) physiology, marking a significant shift in the attention given to the microbiome by neuroscientists. The intricate communication between the gut and the brain is mediated by a multitude of pathways, including immune, enteric, and neural connections. Importantly, the gut microbiota’s influence extends to the CNS, affecting cells such as microglia. Experimental paradigms with germ-free mice and interventions like antibiotics and fecal microbiota transplant demonstrate the role of gut bacteria in neurotransmitter signaling, myelination, synaptic plasticity, neurogenesis, and cognitive functions. This emerging understanding of the gut–brain axis sheds light on the significant impact of the microbiome on CNS health and function [33].

Furthermore, separate sets of experimental and clinical data have also supported the notion that disturbances in gut microbiota and its interaction with the host can contribute to neurodegeneration [34]. Research findings indicate that low vitamin levels (hypovitaminosis) have been associated with numerous human diseases, especially those that tend to emerge with age, including Alzheimer’s disease, cancer, cardiovascular conditions, type II diabetes, multiple sclerosis, and various inflammatory disorders [35]. In this review, we propose that a combined approach involving probiotic and vitamin co-supplementation could be beneficial in preventing and delaying the onset of Alzheimer’s disease.

## 2. Probiotics

The term “probiotic” was introduced by Lilly and Stillwell in 1965 to describe substances generated by one organism that promote the growth of another [36]. Probiotics have a history dating back to at least 1908 when Nobel laureate Eli Metchnikoff suggested that the extended lifespans of Bulgarian peasants might be attributed to their consumption of fermented dairy items [37]. The term “probiotics” has its roots in the Greek language, where it translates to “for life” [38]. According to the Food and Agriculture Organization (FAO, Rome, Italy) and The World Health Organization (WHO, Geneva, Switzerland), the definition of probiotics was established as “living microorganisms”. When provided in sufficient quantities, these microorganisms provide health benefits to the host [39]. An optimal probiotic product should possess characteristics like high cell survival rates, demonstrating resilience to low pH levels, the capability to thrive in the intestinal tract even if the probiotic strain is unable to establish permanent colonization in the gut, adherence to the gastrointestinal epithelium to counteract the flushing effects caused by peristalsis, the ability to interact with or signal the immune cells present in the gut, originating from human sources, a nonpathogenic nature, resistance to processing methods, and proficiency in influencing local metabolic functions [40]. Probiotic products commonly contain microorganisms such as the yeast *Saccharomyces boulardii* or bacteria producing lactic acid, including species from *Lactobacillus* and *Bifidobacterium*. These items fall under the classification of dietary supplements and food products. The bacterial genera commonly used in probiotic formulations include *Lactobacillus*, *Bifidobacterium*, *Escherichia*, *Enterococcus*, *Bacillus*, and *Streptococcus* [41,42,43].

Recommended dosages for probiotic products may vary based on the particular product. However, standard dosing for children generally ranges from 5 to 10 billion colony-forming units per day, while the usual dosage for adults is 10 to 20 billion colony-forming units per day, which are typically administered for a minimum of five days. Probiotic bacteria offer a range of health benefits, including playing a role in diminishing the occurrence of diarrhea linked to antibiotic usage, contributing to a reduction in diarrhea caused by rotavirus infections, showing promise in decreasing the likelihood of the recurrence of superficial bladder cancer, assisting in regulating and enhancing immune system functions, and fostering a balanced immune response, and they have been associated with the improved efficacy of vaccinations, potentially enhancing immune protection, and may help in reducing the colonization of *Helicobacter pylori*, a bacterium linked to stomach-related issues. Probiotics contribute to the management of symptoms associated with irritable bowel syndrome and play a role in regulating LDL-cholesterol levels, thereby contributing to cardiovascular health [44]. There are several ways to prepare probiotics as follows: as capsules, paste, powder, granules, fermented feed, or pelleted feed. It has recently been suggested that inactivated bacteria should be broadly classified as probiotics since they too have probiotic effects, especially the immunological ones [45]. Dairy and its derivatives stand out as valuable reservoirs of probiotics [46]. Over centuries, lactic acid bacteria (LAB), *bifidobacteria*, and various microorganisms sourced from fermented milk have been harnessed for their probiotic potential [47]. In a recent exploration, 148 LAB strains were identified from Kurut, a traditional naturally fermented yak milk in China, where *L. delbrueckii* subsp., *bulgaricus*, and *Streptococcus thermophilus* dominate the microbial landscape [48]. Beyond dairy products, probiotic-rich yeasts and *lactobacillus* strains have been identified in kefir grains, Masai milk, and Koumiss, each capable of influencing immune responses [49,50,51,52]. Current research endeavors focus on traditional fermented products as promising reservoirs of probiotic bacteria. As a dairy product, cheese emerges as a potential carrier for delivering probiotic microorganisms to the human intestine [53]. Yet, the human gastrointestinal tract (GIT) itself serves as a significant source of probiotics. With more than 500 bacterial species residing in the adult human gut, it represents a rich reservoir of beneficial microorganisms [54]. Notably, probiotic strains are not confined to dairy; non-dairy fermented substrates, including fruits (*L. paracasei* and *L. plantarum*) and meat (*L. sakei*, *L. curvatus*, and *Staphylococcus carnosus*) have exhibited metabolic and functional properties akin to those found in the human intestinal milieu, as demonstrated by in vitro experiments [55,56]. Severe side effects are uncommon, and there are no documented interactions with medications [57]. These strains need to exhibit resistance to adverse conditions such as bile, hydrochloric acid, and pancreatic juice. Furthermore, these microorganisms should display attributes like anti-carcinogenic properties, immune system stimulation, reduced intestinal permeability, lactic acid production, and the ability to endure both the acidic environment of the stomach and the alkaline conditions of the duodenum [57].

### 2.1. The Brain–Gut Axis

The brain–gut axis comprises essential elements such as the CNS and other systems. The brain and gut make contact with each other through a million neurons [58,59]. The axis defines a dynamic and mutually influential link uniting the intestinal bacteria and the CNS. The interlinkage occurs in the CNS and the intestine and encompasses a variety of neuroimmune and hormonal mediators, thereby enabling the facilitation of neuroimmune signaling. Scientists have noted a two-way direction flow joining each other, thus indicating that alerts originating in the brain can affect the various functions of the intestine. Conversely, messages emanating from the gut can impact different brain activities. Nerves or hormones produced by endocrine cells, such as 5-hydroxytryptamine-serotonin (5HT), assist the intestine and the CNS in communicating with each other [60,61].

There are a minimum of 12 distinct types of these cells, with subtypes like A, K, and L cells forming subgroups along the intestine, each housing unique combinations of molecules [62]. A, D, and L cells are types of enteroendocrine cells. Specifically, enteroendocrine A cells located in the stomach (corpus) are involved in the release of the Ghrelin hormone. Stimulating the secretion of satiety hormones by A cells has the potential to reduce food intake [63]. D cells are distributed across the entire GIT. In the colon and rectum, they represent the least common type of enteroendocrine cells (EECs). These spindle-shaped cells often feature a slender apical process and a shorter, wider basal extension. Their secretory granules, approximately 150–300 nm and round in shape, contain the secretory product of somatostatin. Somatostatin serves as a primary inhibitory hormone exerting control over both the endocrine and exocrine functions within the digestive system. Additionally, it can stimulate peristalsis in the colon. L cells are present from the duodenum to the rectum, with a lower occurrence before the terminal ileum and with the highest amount being in the rectum. These bottle-shaped cells typically feature an apical process reaching the luminal surface and, at times, a basal process along the basement membrane. Their secretory granules, approximately 200–400 nm and round in shape, contain various peptides including GLP-1, GLP-2, peptide YY, oxyntomodulin, and glicentin. Peptide YY (PYY) is a multifunctional protein that inhibits intestinal motility and gastric emptying, suppressing pancreatic exocrine function and gastric acid secretion while also curbing appetite and promoting mucosal enterocyte proliferation. By delaying gastric emptying and promoting postprandial satiety, GLP-1 contributes to the incretin effect. Mucosal enterocyte proliferation is stimulated by GLP-2. Glicentin suppresses gastric emptying and promotes the growth of mucosal enterocytes. Oxyntomodulin is involved in inhibiting gastric emptying. In summary, these hormones collectively orchestrate a complex regulatory network influencing various aspects of gastrointestinal physiology, including digestion, satiety, and mucosal health [64]. EECs are distributed among gut epithelial cells throughout the entire gut length, boasting over 20 different signaling molecules, which are often co-localized and co-released. When prompted by chemical or mechanical stimuli, these molecules are released, thus entering the systemic circulation. They can then travel to CNS centers that are responsible for ingestive behavior, such as the nucleus tractus solitarius and the hypothalamus. Alternatively, they may act locally, activating nearby afferent vagal terminals in the gut or liver, thereby generating signals to the brain. Critical in this intricate network are the receptors identified on these cells, influencing the regulation of satiety and hunger. Microbial metabolites, such as bile acids and short-chain fatty acids (SCFAs), trigger these receptors to become activated. The complex interplay underscores the influence of gut–endocrine communication on sensations of hunger and fullness [62].

Additionally, various hormones, including the corticotropin-releasing factor (CRF), corticotropin-releasing hormone, adrenocorticotropic hormone, the hypothalamic–pituitary–adrenal axis, and SCFAs, play a critical role in sustaining gastrointestinal use. Establishing this connection between the intestine and the CNS is necessary for maintaining host homeostasis [28,60,61]. In response to stress, on the level of the enteric nervous system, the CRF underscores the substantial role of peripheral pathways in locally regulating intestinal functions. Simultaneously, 5-HT is recognized as the primary biological foundation in the development of mood disorders and a potent regulator in the gastrointestinal system [65]. Additionally, there is evidence pointing to the involvement of serotonergic signaling in the neurobiology of anxiety [66]. The extensive interplay between commensal bacteria and the immune system in the gut is noteworthy. Emphasizing this, it is crucial to recognize that the gut microbiota significantly shapes the development and functioning of immune cells resident in the central nervous system, particularly microglia. In mouse models of conditions like multiple sclerosis and stroke, the influence of gut microbes on key aspects such as autoimmunity, inflammation, and the movement of immune cells has been prominently established. This underscores the substantial impact of gut microbial regulation on the intricate dynamics of CNS immune responses [62,67,68].

### 2.2. Gut Microbiota and Other Neurodegenerative Diseases

Multiple research studies have indicated that microbial dysbiosis contributes to various neurodegenerative disorders. Current study has prioritized investigating the influence of gut microbiota on a range of neurological disorders, including anxiety, schizophrenia, autism, Parkinson’s disease, and Alzheimer’s disease [69]. Extensive research explores the microbiota’s effects on behaviors like depression and anxiety. When germ-free (GF) mice were compared with specific pathogen-free (SPF) mice, it was evident that GF mice displayed decreased anxiety levels and heightened motor activity [70,71]. During periods of stress and anxiety, scientists have observed changes in certain microbial populations, including *Porphyromonadaceae*, *Clostridium*, *Bacteroides*, *Odoribacter*, *Alistipes*, and *Coriobacteriaceae* [72,73,74]. In elderly individuals, the presence of gut dysbiosis coincides with a decline in cognitive and behavioral functions, as well as a reduction in brain volume; these manifestations bear resemblance to the characteristics observed in age-related brain disorders like Parkinson’s disease [75]. In a cohort study involving 72 Parkinson’s disease (PD) patients and 72 healthy subjects, an examination of the fecal microbiome revealed noteworthy findings. PD-affected individuals exhibited a substantial reduction (approximately 78%) in *Prevotellaceae*. *Prevotellaceae* is a major source of mucin, which is a heavily glycosylated protein that serves to safeguard the epithelial lining from pathogens. Additionally, PD patients displayed a significant increase in *Enterobacteriaceae*, which showed a positive correlation with postural instability [76]. Furthermore, *Faecalibacterium* spp. and *Anaerostipes* are also found to be decreased in PD [77,78], as well as *Collinsella* and *Slackia* (*Actinobacteria*), which are also reduced, while the genera *Euryarchaeota* and *Verrucomicrobia* are increased [79] in PD. In patients with Parkinson’s disease and animal models, inflammatory reactions in the colonic tissues are observed. These responses involve an increase in chemokines and proinflammatory cytokines, specifically IL-1β, IL-6, IL-17, interferon-gamma (IFN-γ), and TNF, in addition to enhanced ECG-reactive proliferation and the activation of inflammatory cells. Furthermore, there is a proposal that the gut microbiota and their metabolites may play a role in the development of Parkinson’s disease by influencing neuroinflammation, barrier integrity, and neurotransmitter function [80]. The connection between gut bacteria and multiple sclerosis (MS) is further substantiated by a notable reduction in *Faecalibacterium* concentration in MS patients when compared with that of their healthy counterparts. This decline is significant as it points towards a potential link between alterations in the intestinal microbiome and an increased susceptibility to developing MS. One proposed mechanism involves the production of butyrate, a short-chain fatty acid associated with the expansion of regulatory T cells (Treg), suggesting a plausible pathway through which changes in gut microbiota composition may contribute to a predisposition to MS development. Moreover, individuals with MS undergoing treatment with glatiramer acetate exhibit distinct microbial profiles compared with untreated MS patients. Specifically, there is a reduction in the abundance of *Bacteroidaceae*, *Faecalibacterium*, *Ruminococcus*, *Lactobacillaceae*, *Clostridium*, and other members of the *Clostridiales* class. This observation implies that the therapeutic intervention with glatiramer acetate is associated with alterations in the gut microbiome, suggesting a potential interplay between disease-modifying treatments and the composition of intestinal microflora in individuals with MS [81]. Moreover, when subjected to treatment, individuals with MS experience a reduction in bacteria belonging to the *Sarcina* genus [82]. Evidence in the context of amyotrophic lateral sclerosis (ALS) suggests the presence of intestinal dysbiosis, which is marked by an imbalance in the gut microbial community. Additionally, in rodents expressing the SOD1 (G93A) mutation, there is an observed elevation in intestinal permeability. This finding implies that alterations in the gut microbiota composition and an increase in the permeability of the intestinal barrier are noteworthy features associated with ALS [83]. The *Firmicutes*:*Bacteroidetes* ratio, which is known to undergo changes with age, is a subject of exploration regarding its association with neurodegeneration. However, evidence does suggest a decreased *Firmicutes*:*Bacteroidetes* ratio at the phylum level in ALS. This highlights the complexity of the relationship between the *Firmicutes*:*Bacteroidetes* ratio and different neurodegenerative diseases [84]. During the initial stage of the disease, there is compelling evidence indicating dysbiosis in terms of bacterial composition. Notably, this dysbiosis is characterized by a significant reduction in *Butyrivibrio fibrisolvens* and *Firmicutes*, both of which play a crucial role in producing butyrate. Simultaneously, there is a decline in the expression of proteins involved in tight and adherent junctions, thus leading to an increased permeability of the intestinal epithelium. In ALS, the concentration of *Escherichia coli* also decreases with the additional presence of the polyphenols *Butyrivibrio fibrisolvens* and *Firmicutes* [85]. Moreover, the observed rise in the number of abnormal Paneth cells, which are specialized intestinal epithelial cells with a primary role in detecting microbes and secreting antimicrobial peptides, further contributes to the complex alterations in the intestinal environment. These intricate changes collectively highlight the multifaceted nature of dysbiosis during the early stages of the disease [86]. In addition, research has unveiled disturbances in the gut microbiome and shifts in metabolism in various other neurodegenerative disorders, such as prion diseases and Huntington’s disease [87].

### 2.3. Alteration in the Composition of Gut Microbiota Plays a Role in the Development of Alzheimer’s Disease

**Figure 1:**
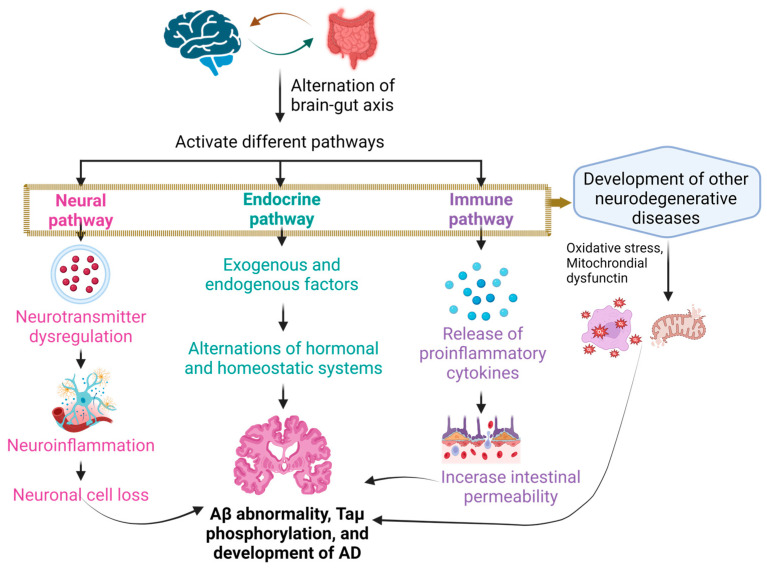
Contributing factors in the development of Alzheimer’s disease. The alteration of the gut–brain axis initiates various pathways, including neuronal, endocrine, and immune pathways, leading to disruptions in neurotransmitters and hormonal regulation. Simultaneously, the release of diverse cytokines contributes to the collective mechanisms fostering the development of Alzheimer’s disease. Ultimately, the multifactorial approach involving these interrelated processes contributes to the onset and progression of Alzheimer’s disease.

Gut bacteria are significantly involved in the pathogenesis of Alzheimer’s disease. Gut bacteria can release substantial amounts of lipopolysaccharide (LPC) and amyloid, both of which play a role in modulating signaling pathways triggering neuroinflammation, activate proinflammatory cytokines such as IL-17A and IL-22, and play a role in the development of Alzheimer’s disease (Figure 1). Moreover, changes in the gut microbiota are closely linked to other factors that are known to be involved in the development of AD, such as type 2 diabetes and obesity [88,89]. The development of Alzheimer’s disease could be impacted by the condition of both the intestinal barrier and the blood–brain barrier (BBB). Changes associated with aging contribute to increased permeability in both barriers. This heightened permeability coupled with disruptions to the BBB and intestinal barrier caused by gut dysbiosis may potentially amplify the entry of pathogens into both the bloodstream and the brain [90,91].

Additionally, in Alzheimer’s disease, various bacterial species can exacerbate the formation of amyloid β plaques. These species include *Mycobacterium* spp., *Salmonella* spp., *E. coli*, *Streptococcus* spp., and *Staphylococcus aureus* [92]. AD patients have shown an increased abundance of Gram-negative bacteria, coinciding with mucosal disruption in response to this dysbiosis [92,93]. Germ-free mice exhibited a notable increase in the hypothalamic–pituitary–adrenal response when compared with mice with healthy gut florae. Reestablishing a balanced gut microbiome in the early stages partially corrected the stress response of the hypothalamic–pituitary–adrenal axis. Intriguingly, the hippocampus and cortex of germ-free mice showed reduced levels of BDNF, a neurotrophin essential for synaptic plasticity and neuronal survival [94].

Furthermore, central nervous system development and behavior are affected by microbiome imbalances. Germ-free conditions impair hippocampal development, alter blood–brain barrier permeability as well as hormone levels, and affect memory, learning, physical activity, and anxiety [95]. If the intestinal barrier breaks, gut bacteria-produced LPS interacts with the Toll-like receptor 4 (TLR4) signaling pathway to activate the immune system. [96]. LPS triggers the inflammatory response of the TLR4 by engaging in interactions with clusters of differentiation 14 (CD14) and myeloid differentiation factor 2 (MD-2). Furthermore, the involvement of CD14 in activating the TLR4 also promotes the inflammatory response associated with Aβ in Alzheimer’s disease [34].

Alterations in the probiotic composition have a noteworthy impact on the progression of Alzheimer’s disease. Studies have confirmed that AD is associated with a marked reduction in the abundance of bacteria from the genera *Verrucomicrobia*, *Actinobacteria*, *Firmicutes*, and *Proteobacteria*. At the same time, there is a consistent trend of the increased presence of the genera *Tenericutes* and *Bacteroidetes* in AD cases [34]. The composition of gut microbiota is under the influence of various external elements like diet, lifestyle, and infections, as well as internal factors such as genetics, metabolites, immune responses, and hormones. This microbial composition, in turn, plays a role in generating neurotransmitters or neuromodulators, including choline, tryptophan, short-chain fatty acids, and hormones like ghrelin and leptin in the GIT, thereby exerting control over the central nervous system (Figure 1) [97]. SCFAs have demonstrated potent capability in vitro to prevent the formation of harmful soluble beta-amyloid aggregates effectively. Moreover, an increasing amount of research indicates that the levels of circulating SCFAs can influence the functioning of the central nervous system. This suggests that SCFAs may play a functional role in regulating amyloidosis, neuroinflammation, and other conditions related to Alzheimer’s disease in the brain [98].

### 2.4. Role of Probiotic Bacteria in Other Neurodegenerative Diseases

Probiotic bacteria play a dual role in influencing the immune responses of the host and fostering a harmonious gut environment by maintaining a balance in the intestinal microflora. The consumption of probiotics has the potential to reestablish a favorable composition of the gut microflora, thus promoting conditions conducive to the thriving of beneficial microorganisms [92]. In a recent study, it was observed that probiotics demonstrated a capacity to diminish oxidative stress, mitigate the presence of pro-inflammatory cytokines, and effectively counteract the overgrowth of pathogenic bacteria in individuals with Parkinson’s disease [99]. Research findings indicate that *Lactobacillus plantarum* PS128 has demonstrated the ability to alleviate the death of nigral dopaminergic neurons and mitigate motor deficits in a mouse model of Parkinson’s disease [100]. *Lactobacillus brevis* and *Bifidobacterium dentium* found in the human microbiota exhibit proficiency in producing γ-aminobutyric acid (GABA) and hold promise for ameliorating abnormalities associated with depression-like symptoms [101,102]. Many neurological and psychiatric disorders are related to changes in the levels of 5-HT and DA (dopamine). To modulate the signaling of these molecules, the complex probiotic VSL-3, comprising eight bacterial strains, engages with mesenchymal stromal cells (hMSCs). This interaction results in a reduction in neurodegeneration and the inhibition of NOD-like receptor protein-3, a mediator of inflammation, without affecting the impacts of hMSCs [103].

The administration of a sole strain, *Bifidobacterium longum*, has shown cognitive enhancement in healthy Balb/c mice. In a distinct scenario involving the adult population, the utilization of a multi-strain probiotic encompassing various species of *Lactobacilli* and *Bifidobacterium* exhibited enhanced cognitive function [104]. In the context of multiple sclerosis, *B. animalis* contributes to enhanced gut barrier function and diminished inflammation through the regulation of permeability in the gut barrier [105]. In amyotrophic lateral sclerosis, *Lactobacillus rhamnosus* is associated with the regulation of behavioral changes resulting in improved behavior and enhanced social interaction. Additionally, *Bifidobacterium longum* contributes to the regulation of stress and anxiety levels, leading to a reduction in symptoms of anxiety and depression [106,107]. From a broader perspective, a variety of probiotics, including *E. coli*, *Lactiplantibacillus plantarum*, *Bifidobacterium pseudocatenulatum* and diverse combinations encapsulated in tablets or capsules, have demonstrated potential benefits in serving as anti-inflammatory and antioxidant agents. Moreover, they exhibit the capacity to modulate the release of anti- and pro-inflammatory cytokines, suggesting a potential role in reducing the likelihood of neurodegenerative conditions in patients [108].

### 2.5. Probiotics Contribute to Potential Therapeutics in Alzheimer’s Disease

Numerous in vivo studies have revealed a significant contribution of probiotic bacteria to enhancing the cognitive function of individuals with Alzheimer’s disease. Presently conducted research indicates that administering probiotics offers neuroprotective advantages, thereby mitigating cognitive impairments and influencing dysbiosis in the gut microbiota. These effects may be associated with pathways involving oxidative stress and inflammation. Several individual strain probiotics have been reported for their potential effectiveness in enhancing memory and improving behavioral activity. *Lactobacillus plantarum* MTCC 1325, a singular strain, demonstrated enhancements in spatial memory, elevated gross behavioral activity, and the development of hyperchromatic nuclear chromatin in the cytoplasm when compared with the results of the control group. Notably, the *L. plantarum* MTCC1325 strain, which is known for its capacity to produce the neurotransmitter acetylcholine, holds promise for reinstating acetylcholine levels in the cortex and hippocampus of mice models with Alzheimer’s disease through probiotic administration [109]. *Lactobacillus acidophilus*, along with *Bifidobacterium bifidum* and *Bifidobacterium longum* NK46, not only protects against Aβ-induced cognitive dysfunction but also mitigates inflammation and immune-reactive gene expressions in the hippocampus of mice models with Alzheimer’s disease. Kobayashi et al. found that probiotic administration suppressed the expression of genes associated with inflammation and oxidative stress in the hippocampus of an AD mouse model [110]. This suggests that the beneficial effects of probiotics may arise from their anti-inflammatory and anti-oxidative properties. Our analysis revealed a significant decrease in certain inflammatory and oxidative biomarkers (hs-CRP and MDA) after the probiotic intervention, potentially contributing to the positive effects observed for individuals with AD or mild cognitive impairment (MCI). However, there were no significant differences in other biomarkers (TAC, GSH, and NO) in the meta-analysis. Considering the variations in intervention conditions, such as flora strains, overall intervention duration, dosage, etc., it is important to note that inflammatory and oxidative stress pathways may not be specific to AD and MCI but could play a significant role in various diseases, especially age-related conditions. Additionally, the limited selection of inflammatory and oxidative biomarkers in this study represents only a portion of the pathophysiology in AD and MCI patients. Therefore, future clinical trials should explore a more comprehensive range of inflammatory and oxidative metabolites to uncover the precise mechanisms behind the positive effects of probiotics on individuals with AD and MCI [111].

Additionally, these probiotics alleviate cognitive decline in 5xFAD transgenic mice by individually modulating the gut microbiota and regulating the nuclear factor-kappa B (NF-κB) activation mediated by LPS [110,112]. Furthermore, enhancements in spatial learning and memory, restoration of synaptic plasticity in the hippocampus, prevention of Aβ peptide accumulation in the hippocampus, reduction in malondialdehyde (MDA) levels in the brain, and an increase in the total antioxidant capacity in plasma were observed for the administration of *Bifidobacterium longum*, *Bifidobacterium bifidum*, and *Lactobacillus acidophilus* [113]. Similarly, *Clostridium butyricum*, *Akkermansia muciniphila*, and *Agathobaculum butyriciproducen* demonstrated significant efficacy in alleviating cognitive deficits. These probiotics also played a role in diminishing microglia-mediated neuroinflammation and Aβ deposition. The positive effects of these probiotics contributed to the improvement in cognitive impairment and a reduction in the Aβ plaque burden in the brains of APP/PS1 mice [114,115,116]. Among potential probiotics for Alzheimer’s disease, *Bifidobacterium longum* (NK46), *Clostridium butyricum*, and the composite SLAB51 emerge as ones that are particularly promising due to their notably positive results. *Bifidobacterium longum*, which is characterized as an anaerobic, non-halophilic, Gram-positive bacterium, naturally inhabits the human gastrointestinal tract [117].

In the context of multi-strain probiotics like SLAB51, comprising *Streptococcus thermophilus*, *Bifidobacterium breve*, *Bifidobacterium longum*, *Bifidobacterium infantis*, *Lactobacillus plantarum*, *Lactobacillus acidophilus*, *Lactobacillus delbrueckii* subsp. *bulgaricus*, *Lactobacillus paracasei*, and *Lactobacillus brevis*, notable effects were observed. These included increased levels of glucose transporters 1 (GLUT1) and glucose transporter 3 (GLUT3) in the CA1 region of the hippocampus, decreased levels of phosphorylated tau aggregation in the brain, elevated HbA1c plasma concentrations, and increased brain expression of the insulin-like growth factor-I receptor (IGF-IRβ), elevated levels of glutathione peroxidase (GPx), glutathione-S-transferase (GST), superoxide dismutase (SOD), and catalase (CAT) were observed in brain homogenates. Conversely, in brain tissues, there was a decrease in 8-Oxoguanine glycosylase (OGG1) and poly-ADP ribose polymerase (PARP). These outcomes suggest a regulatory influence on both brain function and glucose metabolism and brain homogenates [118]. Furthermore, for Aβ-induced Alzheimer’s disease model mice group, the administration of probiotics containing four *Lactobacillus* and *Bifidobacterium* species markedly enhanced memory function and effectively suppressed Alzheimer’s disease-related pathological mechanisms [119]. In a holistic sense, probiotics exert their effects by contributing to the production of neurotransmitters such as ACh and AChE, thereby overseeing the regulation of brain metabolites, modulating brain homogenates, influencing the balance of brain and glucose metabolism, controlling presynaptic neurotransmitters in the brain, and orchestrating the regulation of CREB (element-binding protein) and BDNF [89].

In a study involving elderly individuals with memory complaints, the effects of *Bifidobacterium breve* A1 supplementation on cognitive function were investigated over 12 weeks. The overall scores from baseline showed no significant differences between the group receiving the probiotic and the placebo group. However, a noteworthy distinction surfaced when a detailed analysis was conducted, which was specifically focused on the immediate memory subscale. Interestingly, no significant differences in blood parameters were observed between the individuals taking the probiotic and those in the placebo group. This nuanced examination underscores the significance of considering distinct cognitive domains and emphasizes potential subtleties in how probiotic supplementation may influence cognitive function in elderly subjects [120]. The study revealed that both sole and multi-strain probiotics offer benefits in enhancing cognitive function. These things considered, *Bifidobacterium* and *Lactobacillus* strains emerge as promising candidates for improving cognitive health among AD patients [111].

## 3. Vitamins and Classifications

Vitamins are necessary organic molecules that are either not generated in the human or animal system or are formed at insufficient levels. As a result, they must be consumed through foods or as a precursor [121,122]. Not obtatining enough vitamins may have major health consequences as they are vital for human growth, development, reproduction, and maintenance [123]. Vitamin deficits that persist over an extended period of time might result in malnutrition and serious health problems [124]. It has been determined that there are four fat-soluble vitamins and nine water-soluble vitamins [122]. The structures and functions of vitamins have been described in Table 1.

### 3.1. Fat-Soluble Vitamins

Fat-soluble vitamins, which are often known as fat-soluble vitamins, are soluble in fats and oils as their name suggests. After absorption, fat-soluble vitamins are typically stored in fat for later use. The four fat-soluble vitamins that have been identified are vitamin A, vitamin D, vitamin E, and vitamin K [124,125]. Certain tissues in the body, such as the retina, epithelial linings, immunological cells, brain, nerve tissue, and bones, are among the tissues that highly benefit from the presence of fat-soluble vitamins. Several major clinical issues can arise at any point in the human life cycle if these vitamins are not absorbed or consumed in sufficient quantities [125].

### 3.2. Water-Soluble Vitamins

In terms of both their structural and functional characteristics, water-soluble vitamins are molecules that are essential for the regular functioning of cells, as well as for growth and development. Water-soluble vitamins are excreted and not readily stored in the body [126]. As a general rule, water-soluble vitamins are regarded as a micronutrient, and a lack of them is responsible for developing serious disorders such as neurological disorders, stunted growth, and digestive ailments. The essential water-soluble vitamins are as follows: vitamin B1, vitamin B2, vitamin B3, vitamin B5, vitamin B6, vitamin B7, vitamin B9, vitamin B12, and vitamin C [124,125,126]. As a result of the fact that humans are unable to produce water-soluble vitamins (with the exception of niacin), they need to get these vitamins from exogenous sources through intestinal absorption [125].

**Table 1:** Vitamin structure and functions.

Vitamin	Alternate Name	Year of Discovery	Structure(s)	Functions	Refs.
Vitamin A (Fat-soluble)	Retinol	1916	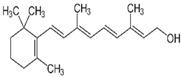	Involved in: Process of vision Reproduction Embryonic development Gene expression Immune function	[121,123,125,127,128,129]
Vitamin D (Fat-soluble)	Calciferol, Ergocalciferol (D2), Cholecalciferol (D3)	1918	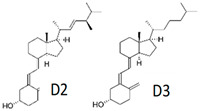	Control of cell division and growth Bone mineralization Regulation of serum phosphate and calcium levels Modulation of the immune system	[121,123,125,129,130]
Vitamin E (Fat-soluble)	α-tocopherol	1922	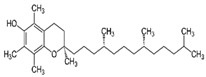	Regulates gene expression. Involved in cell signaling and functions. Possesses antioxidant properties.	[121,123,125,129,131]
Vitamin K (Fat-soluble)	Phylloquinone (K1), Menaquinone (K2), Menadione (K3)	1929	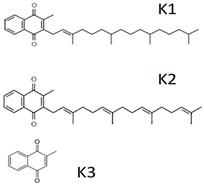	Coagulation of blood. Metabolism of bones.	[121,123,125,128,129,132,133,134]
Vitamin B1 (Water-soluble)	Thiamine	1912	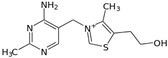	Acts as a cofactor in energy metabolism	[121,123,125,135]
Vitamin B2 (Water-soluble)	Riboflavin	1920	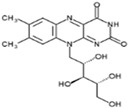	Plays an important role in the respiratory chain and energy production. Possesses antioxidant properties. Promotes iron metabolism.	[121,123,125,136]
Vitamin B3 (Water-soluble)	Niacin, Nicotinic Acid	1936	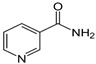	Protects tissues against oxidative damage. Involved in energy storage.	[121,123,125,137]
Vitamin B5 (Water-soluble)	Pantothenic Acid	1931	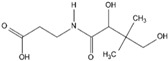	CoA, which is essential for energy metabolism, contains vitamin B5. Involved in hormone production and the immune system of the body.	[121,123,125,138]
Vitamin B6 (Water-soluble)	Pyridoxine and Derivatives	1934	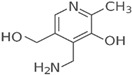	Involved in red blood cell production. Directs several steps in carbohydrate metabolism. Involved in the biosynthesis of neurotransmitters, including GABA, dopamine, and serotonin.	[121,123,125,139]
Vitamin B7 (Water-soluble)	Biotin, Vitamin H	1931	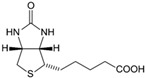	Plays a role in fat deposition in the skin. Required for the proper functioning of insulin. Involved in lactate and pyruvate metabolism.	[121,123,125,140]
Vitamin B9 (Water-soluble)	Folate, Folic Acid	1941	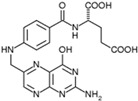	Supports brain health. Regulates blood homocysteine levels. Involved in red blood cell production. Protects against birth defects, especially neural tube defects.	[121,123,125,141]
Vitamin B12 (Water-soluble)	Cobalamin	1926	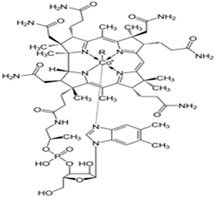	Involved in red blood cell production. Supports brain health. Acts as a co-factor in the process of DNA synthesis. Involved in aerobic energy metabolism.	[121,123,125,142]
Vitamin C (Water-soluble)	Ascorbate, Dehydroascorbate	1912	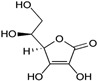	Possesses antioxidant properties. Assists in iron absorption in the intestine. Involved in the production of collagen.	[121,123,125,143]

## 4. Role of Vitamins in Cognitive Function

Multiple circumstantial lines of evidence indicate that elevating vitamin levels may enhance cognitive performance. This evidence indicates that individual vitamins play a crucial role in the physiological and cellular processes that support brain function [122]. In this review, we focus more on vitamins A, B, C, D, E, and K.

### 4.1. Vitamin A

Retinol, retinal (also known as retinaldehyde), retinoic acid, and numerous provitamin (precursor) carotenoids, most notably beta-carotene, are among the chemically related organic substances that are collectively referred to as vitamin A [144]. The primary molecule, retinal, is metabolized into physiologically active retinoid compounds, including retinal, which is the active component of visual pigment, and retinoic acid, which is an intracellular messenger that regulates cell differentiation [145]. Vitamin A can easily enter the brain and become transformed into its bioactive forms. Retinoic acid, the active form of vitamin A, is a powerful signaling molecule in the brain [146,147]. It controls the expression of many genes and influences the growth of new neurons, the survival of existing neurons, and the ability of neurons to change and adapt [147]. Additionally, membrane stability, synaptic plasticity in the hippocampus, and dopaminergic function and transmission are all areas in which it plays a significant role of critical importance. In addition, retinoids and derivatives can directly bind to retinoic acid receptors in parts of the brain that are relevant to cognition, such as the striatum, cortex, and hippocampus [122]. The involvement of retinoic acid in memory is characterized by such a level of complexity that an excess or deficiency in it can lead to comparable impairments in learning behaviors. Optimal learning requires the precise regulation of retinoic acid levels through complex feedback mechanisms [147]. The prefrontal cortex, together with its connections with the mediodorsal thalamus, plays a vital role in cognitive flexibility and working memory. A study conducted on animals has revealed that retinoic acid is necessary for the accurate arrangement of molecular patterns in the prefrontal and motor areas, the development of communication between the prefrontal cortex and the mediodorsal thalamus, and the growth of dendritic spinogenesis inside the prefrontal cortex. These findings demonstrate the crucial involvement of retinoic acid signaling in the formation of the prefrontal cortex and its potential contribution to its evolutionary expansion [148]. Another study conducted on vitamin A-deficient rats revealed that vitamin A deficiency affects postnatal cognitive performance by suppressing the neuronal calcium excitability in the hippocampal regions [149]. Evidence from various studies strongly indicates that vitamin A, specifically through its primary metabolite retinoic acid, continues to have significant effects on brain physiology and behavior in humans. Several animal studies suggest that maintaining an optimal level of retinoic acid is necessary for memory formation and retrieval.

### 4.2. Vitamin B

Vitamin B1 plays a crucial role in energy metabolism. Since the brain is the organ in the body that requires the most energy, a lack of vitamin B1 can have a significant negative impact on its functioning. Additionally, vitamin B1 is essential for the structure, integrity, and development of brain cells. Vitamin B6 is involved in the synthesis process of neurotransmitters, which are the messenger molecules of the brain. Important neurotransmitters that rely on vitamin B6 for their production include dopamine, GABA, and serotonin. Both vitamins B9 and B12 play a crucial role in the development of neurons, their function, maintenance, and overall brain health [122,123,150]. Cognitive impairment is associated with homocysteine, and the metabolism of homocysteine is significantly regulated by folic acid and vitamins B6 and B12. The sole means for endothelial cells to get rid of homocysteine is through the folic acid- and vitamin B12-dependent re-methylation route, which is controlled by the methionine synthase (MS) and methylenetetrahydrofolate reductase (MTHFR) [151]. Homocysteine re-methylation necessitates sufficient quantities of folic acid and B12. On the other hand, vitamin B6 is an essential cofactor in the conversion of homocysteine to cysteine. Elevated homocysteine levels that arise from blocking the conversion process may result from deficiencies in folate, vitamins B6, and B12. These effects can negatively impact cellular, oxidative, and vascular parameters and ultimately lead to a variety of neurodegenerative disorders [122]. A population-based cohort study discovered a correlation between blood folate levels that were normal but low and the likelihood of cognitive impairments in the older population. Additionally, a fall in serum folate levels that were normal but low was connected with the occurrence of dementia. It is possible that maintaining a blood folate content that is higher than 5.9 ng/mL is helpful for cognitive activity [152].

### 4.3. Vitamin C

The majority of species can produce vitamin C from glucose in the liver. However, higher order primates, including humans, rely on obtaining an adequate amount of vitamin C from their diet due to a gradual accumulation of mutations over time [153]. A steep concentration gradient facilitates the delivery of vitamin C to the brain where it accumulates at high concentrations in regions dense with neurons, including the cerebellum, hippocampus, and cortex. Vitamin C is involved in neuronal development, neuromodulation, and the production of tyrosine, carnitine, and catecholamine neurotransmitters [122]. Hypoxia-inducible factor-1α (HIF-1α) plays a role in the development of neurons, maintaining the balance of oxygen levels and promoting the growth of blood vessels. The regulation of this HIF-1α is dependent on vitamin C-related hydroxylation and a successive process of degradation. Therefore, deficiency in vitamin C may lead to elevated levels of HIF-1α, which can disrupt the normal development of blood vessels. This is especially crucial during fetal growth and in cases of brain injury restoration [153]. The brain is very susceptible to oxidative damage due to its abundance of polyunsaturated fatty acids and its elevated cellular metabolism. Multiple investigations involving cells and animal models have shown evidence of the significant importance of vitamin C in the brain. Vitamin C functions as a potent antioxidant and helps eliminate reactive oxygen species (ROS) [153]. Vitamin C plays an essential role in the development of neurons and the synthesis of myelin [154,155]. It is also engaged in the transmission of signals in the central nervous system through neurotransmitters. Presynaptic reuptake of glutamate appears to be another neuromodulatory function of vitamin C. Excess extracellular glutamate causes excitotoxic damage, causing the N-methyl-d-aspartate (NMDA) receptor to become hyperpolarized and initiate subsequent damage to neurons. However, vitamin C can prevent this from happening. Additionally, vitamin C has been demonstrated to prevent glutamate from binding to the NMDA receptor, directly preventing glutamate from overstimulating neurons [153]. A population-based cross-sectional study demonstrated that there is a strong correlation between the levels of vitamin C in the blood and performance on tasks related to working memory, attention, focus, delayed and total recall, decision speed, and recognition performance [156].

### 4.4. Vitamin D

The primary compounds that hold significant importance in humans are vitamin D3 (cholecalciferol) and vitamin D2 (ergocalciferol). In humans, only 20% of vitamin D is obtained from diet; the majority of it is created in the skin through UVR irradiation. Vitamin D obtained through dietary sources or synthesized by the skin is physiologically inert. The activation process involves two hydroxylation processes catalyzed by enzymes, with the first occurring in the liver and the second in the kidneys [157]. In the liver, ergocalciferol becomes 25-hydroxyergocalciferol, while cholecalciferol becomes calcifediol (25-hydroxycholecalciferol). To assess an individual’s vitamin D status, serum levels of these two vitamin D metabolites, which are often known as 25-hydroxyvitamin D or 25(OH)D, are examined [158]. The kidneys further hydroxylate calcifediol to generate calcitriol (1,25-dihydroxycholecalciferol) or 1,25(OH)2D, which is the physiologically active form of vitamin D [159]. To reach tissues and organs, vitamin D enters the bloodstream and attaches to chylomicron or vitamin D-binding proteins. In addition to preserving calcium homeostasis, vitamin D is essential for the immune system [160] and cognitive functions [161]. Vitamin D exerts its effects by binding to its specific receptor VDR (vitamin D receptor), a nuclear hormone receptor located in the central nervous system. VDRs are found in neurons and glia cells, particularly in the amygdala, thalamus, and nucleus accumbens, as well as in the temporal, cingulate, and orbital cortices—all areas that are crucial to the maturation of brain functions. Moreover, the varied location of VDRs throughout the brain implies that vitamin D might have a role in the differentiation of stem cells and the proliferation of neurons [162]. Experimental investigations conducted on animal models or cells involving vitamin D have significantly supported the association between vitamin D and neuroprotection. Vitamin D plays a variety of roles in the neurological system, such as regulating oxidative stress mechanisms, controlling the generation of neurotrophic factors, releasing neurotransmitters, and altering inflammatory responses [161]. Vitamin D has demonstrated the ability to control crucial neurotrophic factors in the brain [122], including nerve growth factor (NGF), increase the expression of neurotrophin 3 (NT3), and enhance the glial cell line-derived neurotrophic factor (GDNF). Vitamin D may also influence neural plasticity mechanisms such as the growth of axons. Recent research has demonstrated that vitamin D has a neuroprotective effect, particularly through stimulating the production of calcium-binding proteins such as parvalbumin. Vitamin D has also been shown to impede the production of an inducible nitric oxide synthase, an enzyme that is activated in neurons and non-neuronal cells under ischemic conditions or in neurodegenerative disorders [163].

### 4.5. Vitamin E

The biological activity of vitamin E is not just confined to its antioxidant qualities. Instead, many forms of vitamin E have an impact on gene expression, cell proliferation, membrane-bound enzymes, cellular signaling, the regulation of inflammation response, and an array of molecular pathways [164]. Several animal and human studies have confirmed the significant role of vitamin E on cognitive function. In an animal study, it was concluded that a lack of vitamin E caused Purkinje neurons, which are the main output regulators of the cerebellar cortex, to undergo cellular atrophy and show less dendritic branching. Deficits in motor coordination and cognitive abilities coincided with the structural and functional decline caused by vitamin E insufficiency. In the same study, the fear conditioning test was significantly inadequate in vitamin E-deficient tocopherol transfer protein (TTP) null mice. As TTP regulates α-tocopherol levels and distribution throughout the body, it is regarded as the “gatekeeper” of vitamin E. Mutations in the TTPA gene result in the deficiency in vitamin E and lead to the development of ataxia, thereby confirming the fact that TTP is crucial for the maintenance of vitamin E status in the body. The authors concluded by highlighting the vital role of vitamin E in the brain’s amygdala area, which is believed to regulate memory formation and emotional reactions. Crucially, prompt α-tocopherol administration prevented these functional deficiencies [165]. A cross-sectional study discovered that a decreased incidence of cognitive impairment in older adults was correlated to a larger consumption of dietary vitamin E [166].

### 4.6. Vitamin K

Vitamin K plays a crucial role in the process of blood clotting. Recent studies suggest that this vitamin may provide significant advantages in both preventing and treating bone and vascular diseases as well [167]. Studies have revealed intriguing but inconclusive indications of a direct association between vitamin K levels and cognition [168]. Several human studies seem to demonstrate similar results. A cross-sectional study (CLIP) investigated the effects of dietary vitamin K intake in older adults. It concluded that a higher dietary phylloquinone intake was associated with enhanced cognitive function and improved behavior in elderly individuals [169]. Another study found that the consumption of vitamin K through diet, specifically that from vegetables, showed a negative correlation with the risk of cognitive decline in older individuals [170].

## 5. Role of Vitamins in AD Pathogenesis

It is well documented that the formation of Aβ plaques and neurofibrillary tangles are the main features of AD. Apart from the Aβ and tau hypotheses, researchers have developed several possible hypotheses for this complex disease including oxidative stress [171], neuroinflammation [172], and hyperhomocysteinemia [173], considering these features as indicators of potential risk factors for AD. Vitamin supplementation has been found to reduce Aβ [174,175], oxidative stress [176,177], neuroinflammation [178], and homocysteine levels [179]. Considering the evidence, it is certain that the beneficial effects of vitamins are due to their antioxidant and anti-inflammatory properties, as well as enzymatic activities.

Patients with AD exhibit decreased levels of vitamin A in their serum and plasma. Necessarily, it was demonstrated that the transportation and functioning of retinoic acid were impaired in the AD brain [180]. Therefore, vitamin A deficiency is considered to be an important factor in the development of AD. Oxidative stress, neuroinflammation, mitochondrial dysfunction, and neurodegeneration leading to the gradual progression of AD may occur as a consequence of impaired retinoic acid signaling. In microglia and astrocytes, retinoids suppress the production of chemokines and neuroinflammatory cytokines, which are triggered in AD. Retinoids induce transcription of their target genes through interaction with retinoid receptors including retinoid X receptors and retinoic acid receptors. Stimulating retinoid X receptors and retinoic acid receptors slows down amyloid-β accumulation, demotes neurodegeneration, and prevents the development of AD [181]. A study conducted on APP/PS1 mice revealed that a deficiency in vitamin A worsened impairments in behavioral learning and memory. It also resulted in increased levels of both Aβ40 and Aβ42 in the brain and gut, decreased the levels of retinol in the liver and serum, reduced the transcription of receptors and enzymes that are related to vitamin A in the cortex, enhanced the production of beta-site APP-cleaving enzyme 1 (BACE1) and phosphorylated tau in the cortex, and reduced the expression of GABA and BDNF receptors in the cortex region of the brain [182]. In another study, the deficiency in prenatal marginal vitamin A enhanced the activity of beta-site APP-cleaving enzyme 1 (BACE1), thereby leading to an increased generation of Aβ and the formation of neuritic plaques. This deficiency also dramatically worsens memory impairments in an AD mouse model [183].

Among vitamin B family members, vitamins B1, B6, B9, and B12 are predominantly associated with cognitive function and overall brain health. Vitamin B1 (thiamine) is a fundamental vitamin that plays a significant role in energy metabolism. Thiamine-dependent enzymes play a crucial role in the process of glucose metabolism. In AD patients who also suffer from thiamine deficiency, these thiamin-dependent enzymes are shown to be decreased [184]. As discussed earlier, homocysteine exerts direct neurotoxic effects on the neurons of the central nervous system. An inadequate amount of vitamin B6 is linked to elevated blood homocysteine levels as it is a necessary cofactor for homocysteine re-methylation [185,186]. Folate deficiency triggers various pathophysiological alterations that are believed to have a role in the development of AD. These changes include mitochondrial dysfunction leading to oxidative stress, disruption of calcium homeostasis, impairment of neurons and synapses, and the buildup of Aβ and hyperphosphorylated tau. The distinctive characteristics of insufficient folate make it highly likely to have a long-term impact on the progression of AD [187]. Vitamin B12 (cobalamin) is essential for brain and nervous system function.

Additionally, it facilitates myelin production. Vitamin B12 deficiency is one of the most common causes of hyperhomocysteinemia, which increases the risk of dementia, especially that of AD. High vitamin B12 levels prevent AD-related brain atrophy and cognitive deterioration [188].

Over the years, vitamin C has been discovered to have numerous favorable effects on neurodegeneration, particularly in AD. The primary mechanisms responsible for the neuroprotective effects of ascorbic acid include its ability to scavenge reactive oxygen species (ROS) [189,190], modulate neuroinflammation, and reduce the fibrillation of Aβ peptides. The existence of metal binding sites leads to the presence of copper, zinc, and iron in Aβ plaques. Metals can influence the structure of Aβ, speeding up the process of fibrillation and increasing the cytotoxicity of Aβ. Vitamin C is also involved in the chelation of ions, including zinc, copper, and iron [190]. Increasing in vivo data substantiates the involvement of ascorbic acid in mitigating factors associated with the development of AD. However, human studies have shown inconclusive outcomes.

Another important factor in the pathophysiology of AD is vitamin D. The pathogenesis of AD involves a number of proteins, and vitamin D affects the regulation of these proteins both directly and indirectly. For instance, in AD, the APP is linked to the development of Aβ plaques, neurodegeneration, and cytotoxicity. By inhibiting the production of APP and triggering the phagocytosis of Aβ peptides, vitamin D plays a beneficial effect. The RyR (ryanodine receptor) protein is linked to aging-related memory loss and cognitive decline. Vitamin D prevents the sensitization of RyR protein in an indirect manner. The PTEN protein is thought to be responsible for inhibiting the mTORC (mammalian target of rapamycin) and reducing the aggregation of Tau. Vitamin D increases the expression of the PTEN protein. Vitamin D receptors suppress the transcription of the *APP* gene, and vitamin D increases the expression of the vitamin D receptor protein. Another protein, which is called RAGE (receptor for advanced glycation end products), is found to be associated with increased oxidative stress, the increased formation of neurofibrillary tangles, and neurodegeneration. Vitamin D potentially suppresses the expression of RAGE [191]. In several studies, the biologically active form of vitamin D has been proven to have neuroprotective properties, for instance, that of clearing amyloid plaques, which is a defining feature of AD. In both Europe and the US, correlations have been found between low vitamin D levels and AD [192]. It is now very well documented that in comparison with matched controls, people with AD have reduced circulating vitamin D concentrations. Furthermore, polymorphisms in the megalin or vitamin D receptor genes have been linked to a higher risk of AD or cognitive decline in several genetic investigations [161].

It is well established that malondialdehyde concentrations, a marker of lipid peroxidation, are markedly higher in AD patients. Furthermore, neurons sustain significant harm from both ROS and RNS. Treatment with vitamin E in rat neural cells reduced oxidative stress indicators and prevented the formation of Aβ-associated ROS [193]. A study on rats has shown that vitamin E deficiency is connected with the expression of several genes that are associated with the initiation and subsequent progression of AD [194]. Proinflammatory cytokines (including IL-1, IL-6, and TNF-α) have been found in higher concentrations in the cerebrospinal fluid and brain tissue of AD patients. Subsequently, both TNFα and IL-1 can boost the expression of the amyloid precursor protein and Aβ peptides [195]. Therefore, it is rational to speculate that anti-inflammatory agents, such as vitamin E, may lower the risk or delay the progression of AD [195]. Based on the findings from an animal study, a deficiency in vitamin E was associated with the accumulation of Aβ due to reduced clearance from both the brain and blood [196]. Numerous investigations revealed that AD patients had different vitamin E levels, with higher brain concentrations and lower plasma levels, therefore suggesting a compensatory response to oxidative damage. Moreover, vitamin E promotes the activation of PP2A (protein phosphatase 2A), which is an enzyme noted to be diminished in AD patients yet essential in preserving the proper balance of tau proteins [164].

**Figure 2:**
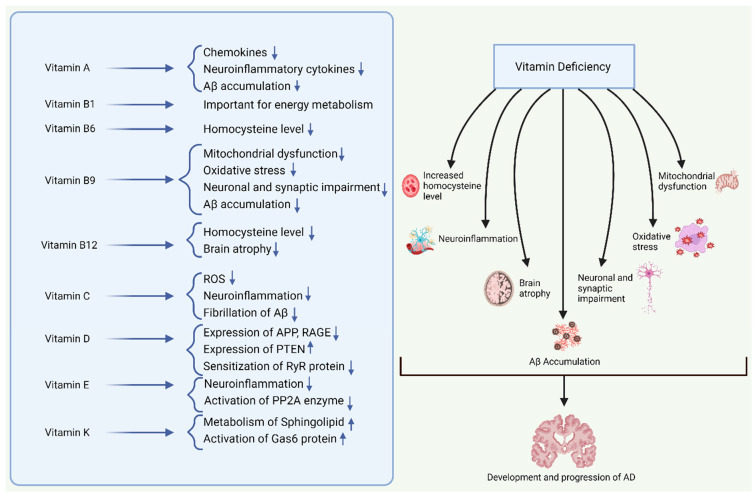
Vitamins play numerous critical functions in the brain. A deficiency in vitamins may lead to a different pathogenesis related to AD, involving increased homocysteine levels, neuroinflammation, mitochondrial dysfunction, oxidative stress, neuronal and synaptic impairment, brain atrophy, and Aβ accumulation. All these pathological features alone and/or cumulatively contribute to the development and progression of AD. In this figure, ‘↑’ denotes increased functional activity/level, ’↓’ denotes decreased functional activity/level.

Mounting evidence suggests that vitamin K plays a crucial role in brain function by participating in the metabolism of sphingolipids and activating the Gas6 protein, which is dependent on vitamin K [197]. Vitamin K insufficiency, which mostly affects the functioning of the vitamin outside the liver, is prevalent among elderly individuals, both males and females [198]. All these findings lead to the hypothesis of a correlation between vitamin K deficiency and the pathogenesis and progression of AD. Different functions of vitamins in AD pathogenesis have been summarized in (Figure 2).

## 6. Role of Vitamin Supplementation in AD Prevention

### 6.1. Vitamin A Supplementation and AD

The use of vitamin A supplementation as a therapeutic agent has been extensively suggested for neurodegenerative disorders including AD [147]. As previously stated, a small deficit in vitamin A encourages the development of the beta-site APP-cleaving enzyme 1 (BACE1)-mediated Aβ and the formation of neuritic plaques. This deficiency also worsens memory impairments in mice with AD. Administering an appropriate amount of vitamin A as a supplement successfully reversed the memory impairments caused by the deficiency in vitamin A in mice [183]. Another study conducted in a 3xTg-AD mouse model suggests that the hippocampus-dependent short-term memory of these mice is preserved with the administration of vitamin A supplementation [199]. Supplementing middle-aged rats with a combination of EPA/DHA (Eicosapentaenoic acid/Docosahexaenoic acid) and vitamin A had a positive impact on reference memory but did not affect working memory [200]. Insufficient research has been carried out to determine the importance of vitamin A supplementation in humans. Further research is necessary to determine the association between vitamin A and memory functions as well as the possible advantages of vitamin A supplementation in a clinical setting. It is substantial to specify that vitamins D and A have a similar type of receptor, which may make supplementing them problematic. Precisely, the nuclear VDR creates homodimers with retinoid X receptor that function as a particular receptor for 9-cis retinoic acid, or it heterodimerizes with nuclear receptors of the retinoid X receptor family and binds to VDREs (vitamin D response elements). Since retinoid X receptor proteins are known to be partners for certain other nuclear receptors, it is possible that the vitamin D and A ligands counteract each other’s effects when there is an excess of vitamin A, which could reduce the effectiveness of vitamin D supplementation [201]. Increased vitamin A intake and elevated serum retinol levels have been linked to an increased risk of fracture and bone fragility in both rodents and humans [202].

### 6.2. Vitamin B Supplementation and AD

Studies on both humans and animals have examined the effects of vitamin B intake either alone or in combination. When B vitamins (choline, folate, B6, and B12) are administered together, the hypoxic memory deficiencies of mice can be effectively improved. Both the blood levels of homocysteine and the excess phosphorylation of the tau protein linked to AD were markedly lowered by this supplement. This is achieved by increasing the activity of inhibitory Ser9-phosphorylated GSK-3β [203]. In a study involving rats, it was revealed that the simultaneous supplementation of folate and vitamin B12 significantly reduced the retinal Aβ accumulation and hyperhomocysteinemia-induced tau hyperphosphorylation at several sites related to AD [204]. According to a recent meta-analysis, while supplementation with vitamin B lowers plasma homocysteine levels, which is a known risk factor for dementia and cognitive impairment, treating adults with and without pre-existing cognitive dysfunction did not improve cognitive functioning [205]. A randomized, single-blinded, placebo-controlled trial investigated the effects of folic acid and vitamin B12 supplementation in AD patients. It concluded that this combined supplementation showed positive therapeutic effects in AD patients [206]. Another study was conducted using a similar combination of vitamin supplementation, vitamin B9, and vitamin B12 on mildly cognitively impaired patients over a period of 24 months. This study concluded that this combined supplementation improved cognitive function at the 24th month in patients with atrophy ratios higher than the median [207]. Supplementation with B vitamins (folic acid 0.8 mg, vitamin B6 20 mg, vitamin B12 0.5 mg) can decelerate the shrinkage of particular brain regions that play a crucial role in the progression of AD and are linked to a deterioration in cognitive function. The authors stated that B vitamins reduce the levels of homocysteine, which in turn results in a reduction in gray matter atrophy, therefore slowing down the decline in cognitive function [208].

### 6.3. Vitamin C Supplementation and AD

Vitamins that exert antioxidant properties are currently receiving a lot of attention as a potential treatment option for AD. The supplementation of antioxidants vitamin C, vitamin E, and selenium in rats increased the levels of brain membrane phospholipids and synaptic proteins, which are indirectly associated with synaptogenesis [209]. Vitamin C reduced the production of Aβ oligomers and behavioral deterioration in a mouse model of AD after six months of treatment. The reduction in Aβ oligomerization significantly reduced oxidative damage in the brain and the ratio of soluble Aβ42 to Aβ40 [210]. Supplementation with a large amount of vitamin C decreases the accumulation of amyloid plaques. It improves the degenerative alterations in the brain of AD mice, leading to improvement in blood–brain barrier disruption and changes in mitochondrial function [211]. The effect of vitamin C supplementation has been studied in humans as well. A meta-analysis of 12 human studies indicated that supplementation with vitamin C can be a viable technique for the prevention and treatment of AD; however, the exact mechanistic role could not be elucidated [212]. A clinical trial with AD patients revealed that administering a combination of 400 IU of vitamin E and 1000 mg of vitamin C to individuals with AD resulted in a significant rise in the levels of both vitamins in the cerebrospinal fluid (CSF) and the plasma after one month of supplementation. Consequently, the vulnerability of CSF and plasma lipoproteins to oxidation was notably reduced. This study suggested that combination therapy could be beneficial over monotherapy of vitamin supplementation with antioxidant properties in terms of delaying the progression of AD [213]. On the contrary, another clinical trial concluded that the administration of vitamins C and E for one year did not have a substantial impact on the progression of AD [214].

### 6.4. Vitamin D Supplementation and AD

Data from animal models on the effect of vitamin D supplementation demonstrate improvements in memory and cognitive function, as well as a reduction in several AD pathology markers. In a study of aging rats, supplementation of high vitamin D3 (10,000 IU/Kg/day) over a period of 5–6 months resulted in the prevention of cognitive decline and improvement in spatial learning and memory [215]. Vitamin D supplementation of 42 I.U./Kg for 21 days using a subcutaneous injection resulted in the modulation of age-related increase in the pro-inflammatory condition and the burden of Aβ in male rats [216]. In a study involving a mouse model of AD, the authors reported that vitamin D supplementation reduced the load of Aβ, increased the NGF levels along with astrocytic reactivity, and reduced the levels of TNFα in the brain [217]. In recent years, several human studies have been performed to examine the effects of vitamin D supplementation on cognitive function and AD. A randomized controlled trial evaluated the effect of high-dose vitamin D2 in patients with mild–moderate AD and did not find any beneficial effects on cognition [218]. In another randomized, double-blind, placebo-controlled trial, it has been highlighted that administering a daily dose of 800 IU of vitamin D orally for 12 months may enhance cognitive performance and reduce Aβ-related biomarkers in older patients diagnosed with AD [219].

### 6.5. Vitamin E Supplementation and AD

Supplementation with vitamins E and C was found to be associated with a decreased risk of AD and all-cause dementia in an observational study [220]. In a clinical trial, supplementation with vitamin E in combination with carotenoids and omega-3 fatty acids resulted in positive outcomes, reducing the severity of the disease in AD patients [221]. A population-based cohort study highlighted that vitamin E supplementation for a longer period exerts a beneficial effect on AD-related neuropathologic changes. This finding supports further investigation to prevent AD with vitamin E supplementation [222]. On a different note, earlier on, a randomized clinical trial of vitamin E supplementation of 600 IU in older women did not show cognitive improvement. This could be partly due to the selection of subjects as the subjects were generally healthy women [223].

### 6.6. Vitamin K Supplementation and AD

Insufficient clinical research involving human subjects exists to elucidate the correlation between vitamin K supplementation and the amelioration of AD. Nevertheless, findings from animal experiments have demonstrated that vitamin K supplementation can reduce neuronal deterioration and cognitive loss [224].

Based on the above finding, we concluded that supplementation with several vitamins either alone or in combination has been shown to offer beneficial effects in managing the symptoms of AD. Combinations of vitamins with distinct properties (For example, vitamin C for antioxidant properties, vitamin E for anti-inflammatory properties, vitamin B for reducing hyperhomocysteinemia) could be a reasonable assumption to have the most beneficial effects.

## 7. Role of Vitamins in Other Neurodegenerative Disorders

Parkinson’s disease (PD) is a neurological disorder characterized by involuntary or uncontrolled movements, including stiffness, shaking, trouble balancing, and proper coordination. The loss of dopaminergic neurons in the middle brain area is the root cause of PD, which manifests itself in a variety of motor and non-motor symptoms. The elderly bear a disproportionate share of the burden of this illness. At the current time, there are no effective medications that can halt the onset or slow the progression of Parkinson’s disease [124]. The neuropathology of PD is distinguished by the gradual demise of dopaminergic neurons in the substantia nigra pars compacta of the midbrain, as well as the formation of Lewy Bodies within neurons. These aggregates are composed of α-synuclein proteins, which result in the death of neurons and subsequently impair the synthesis of dopamine [225]. Experimental and clinical evidence strongly supports the significant involvement of excitotoxicity, inflammation, apoptosis, mitochondrial dysfunction, and oxidative stress in the development of Parkinson’s disease.

Treatment for PD may benefit from the antioxidant qualities of vitamins and their biological roles in controlling gene expression [226]. As mentioned earlier, an important contributor to the onset and progression of Parkinson’s disease is oxidative stress. Vitamin A and its derivatives, like retinoic acid, have potent antioxidant properties [124]. Nicotinamide, the active form of niacin, is a precursor to NADH and NADPH. Through metabolic pathways, nicotinamide biosynthesizes NAD. In ATP synthesis, the mitochondrial complex I needs NADH. Nicotinamide also reduces oxidative stress, thus protecting neurons. Deficiency in NADH is common in patients with PD. Vitamin C inhibits oxidative stress, reduces lipid peroxidation, and scavenges free radicals, supporting nervous system function and antioxidant function. Even though vitamin C has several potential benefits for PD, the serum level of vitamin C in PD patients is contentious. Contrarily, numerous studies found no evidence that vitamin C supplements reduce PD risk [226]. Extensive research has been conducted on the significance of vitamin D in Parkinson’s disease. Reduced levels of vitamin D may potentially cause the death of dopaminergic neurons, which in turn contributes to the development of Parkinson’s disease [225]. One of the prospective therapeutic agents for PD is vitamin E, which, in addition to its powerful antioxidant effects, can also prevent the loss of dopaminergic neurons [226].

ALS is a widely recognized neurodegenerative disorder marked by the gradual deterioration of motor neurons. Divergent findings on the impact of vitamins on amyotrophic lateral sclerosis have been documented. A recent cohort study involving patients from China indicated that the patients had elevated levels of vitamins A and E and decreased levels of vitamins B2, B9, and C in comparison with control subjects. Furthermore, they observed that higher levels of vitamins A and E and lower levels of vitamins B2, B9, and C were linked to a heightened chance of developing ALS. Furthermore, the levels of serum vitamin C were shown to be lower in individuals with early onset ALS in comparison with those with late-onset ALS [227]. Numerous investigations have looked into the effects of vitamin D as a possible ALS therapy. However, no evidence supporting the impact of vitamin D on the prognosis or treatment of ALS was discovered in a recent systematic review and meta-analyses [228].

## 8. Synergistic Effects of Probiotics and Vitamin Co-Supplementation

Numerous studies have demonstrated the synergistic effects of combinational therapies across various diseases. Co-supplementation strategies consistently outperformed their individual counterparts, leading to a range of health benefits. These advantages encompassed reduced disease severity, enhanced mental health, improved metabolic parameters, especially improved insulin sensitivity and dyslipidemia, attenuated inflammation, heightened antioxidative capacity, and a decreased reliance on healthcare resources. Noteworthy findings emerged from studies exploring the combined benefits of probiotics and vitamin D in chronic schizophrenia. The results indicated a significant enhancement in plasma total antioxidant capacity (TAC) and a significant reduction in serum high-sensitivity C-reactive protein (hs-CRP) levels and plasma MDA [229]. Vitamin D is linked with lower inflammatory biomarker levels and also has positive effects on reducing oxidative stress and inflammation. The impact of probiotics on oxidative stress and inflammation has also been reported in many studies. The study suggests a potential synergistic effect of combining vitamin D with probiotics, thus leading to significant improvements in oxidative stress and inflammation markers in individuals with chronic schizophrenia [230]. Recent studies highlighted the potential of probiotic treatment to increase vitamin D levels, enhance vitamin D receptor (VDR) expression, and activate VDR activity in the host. Probiotic strains, such as *L. reuteri* NCIMB 30,242 and VSL#3, have demonstrated the ability to positively impact vitamin D absorption and modulate nuclear receptor signaling pathways. Specifically, probiotics like LGG (*Lactobacillus rhamnosus*) and *Lactobacillus plantarum* (LP) were found to increase VDR protein expression and transcriptional activity, leading to the elevated expression of VDR target genes like *cathelicidin* [231]. The co-supplementation with vitamin D, probiotics, and calcium over six weeks in postmenopausal osteopenic women suggested a potential role in suppressing bone resorption and turnover [232].

The collaborative impact of *Levilactobacillus brevis* IBRC-M10790 and vitamin D3 on Helicobacter pylori-induced inflammation is noteworthy. When vitamin D3 is combined with the probiotic strain, particularly live *L. brevis* and its cell-free supernatant (CFS), there is a more effective reduction in the expression of pro-inflammatory cytokines such as IL-6, IL-8, IFN-γ, and TNF-α in AGS cells. Additionally, the combined action of vitamin D3 and *L. brevis* demonstrates an additive effect in preserving the integrity of the epithelial barrier, as evidenced by an increase in the expression of the tight junction protein ZO-1. Furthermore, this synergistic combination holds promise in potentially reducing *Helicobacter pylori* adherence to AGS cells [233]. Research has indicated that administering a combination of vitamin D and probiotics to women with polycystic ovary syndrome (PCOS) over 12 weeks yielded positive outcomes in various aspects. This co-administration demonstrated favorable effects on mental health parameters, as well as improvements in serum total testosterone, hirsutism, hs-CRP, plasma total antioxidant capacity, glutathione, and MDA levels [234]. Recent research has demonstrated that incorporating probiotic-fortified yogurt or vitamin D-fortified yogurt into daily consumption during a 10-week calorie restriction period leads to an increase in anorectic peptides, specifically GLP-1, without affecting ghrelin levels. The rise in anorectic gut hormones, which are crucial for sustained weight loss and a sense of satiety, contrasts with the potential hindrance to sustained weight loss associated with an increase in ghrelin during low-calorie diets. Such dietary practices are likely to enhance the microbiota–gut–brain axis, thereby influencing homeostasis and daily intake regulation [235]. The pathophysiology of Alzheimer’s disease is intricately connected with the gut microbiome and deficiencies in various vitamins. Research indicates that the interplay between nutrition and mental health involves complex interactions within the gut, thereby influencing neurotransmitter and hormonal pathways that, in turn, impact brain function [89,236,237]. Building upon previous studies [230], a hypothesis emerges that co-supplementation with probiotics and vitamins could yield heightened anti-inflammatory responses, increased total antioxidant capacity (TAC), and decreased high-sensitivity C-reactive protein (hs-CRP) serum levels, along with improved cognitive function. This suggests a potential avenue for interventions targeting both the gut and nutritional aspects to positively influence Alzheimer’s disease-related parameters.

**Figure 3:**
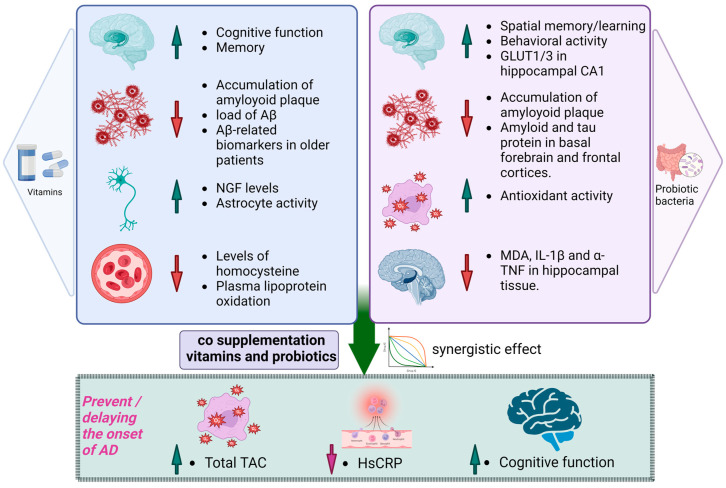
Probiotics and vitamins play distinctive roles in maintaining health, with probiotics supporting gut health and vitamins contributing to various physiological functions, including cognitive wellbeing and others. Combining probiotics and vitamins in a supplement may result in a synergistic effect, potentially leading to enhanced health benefits, particularly in the prevention and delaying the onset of Alzheimer’s disease. In this figure, ‘↑’ denotes increased functional activity/level, ’↓’ denotes decreased functional activity/level. TAC—total antioxidant capacity, hs-CRP—high-sensitivity C-reactive protein.

However, to substantiate this claim further, additional research endeavors will be imperative. As depicted in (Figure 3), there are discernible similarities and divergent effects observed in the impact of vitamins and probiotics. Our proposition posits that a synergistic enhancement of health benefits could be achieved through the co-supplementation of vitamins and probiotics, thereby leveraging their complementary effects. Further investigations are warranted to thoroughly explore and validate the potential synergies between vitamins and probiotics, thus paving the way for a more comprehensive understanding of their combined health-promoting effects.

## 9. Safety and Tolerability

Probiotics and vitamins have been utilized without safety concerns for an extended period. However, the reporting of safety outcomes in published clinical trials is inconsistent. A 2011 report from the Agency for Healthcare Research and Quality highlighted that existing probiotic clinical trials show no evidence of heightened risk [238]. Various probiotic bacteria, including *Lactobacillus acidophilus*, *Lactobacillus rhamnosus*, *Bifidobacterium longum*, and *Streptococcus thermophilus*, were examined in diverse populations encompassing both children and elderly patients. Notably, no indications of toxicity were observed across these studied probiotic strains in these specific demographic groups [239,240,241].

Furthermore, some of them have been incorporated into animal studies focusing on Alzheimer’s disease as well [89]. Nevertheless, the report also emphasized that the current literature lacks the sufficient depth to address safety questions related to probiotics in intervention studies confidently. In theory, probiotics have the potential to induce four distinct types of side effects as follows: systemic infections, adverse metabolic activities, heightened immune stimulation in susceptible individuals, and gene transfer. Furthermore, certain individuals may also encounter minor gastrointestinal symptoms. Critics argue that a wealth of evidence, including the extensive history of safe probiotic use, data from clinical trials, and findings from animal and in vitro studies, collectively supports the assumption that probiotics are generally safe for the majority of populations [238]. Vitamins are extensively examined substances in clinical studies for AD. Their safety and efficacy evaluations commenced in the 1990s and are currently ongoing [242]. High doses of vitamin B (folate 5 mg, vitamin B6 25 mg, and vitamin B12 1 mg) were used in a controlled, randomized trial to assess the safety and effectiveness of B vitamin supplementation in AD. Depression-related adverse events were more prevalent in active treatment. However, the adverse event finding was marginally significant and merely showed a trend toward support [243].

## 10. Discussion

The distinctive features of AD involve the widespread presence of plaques primarily composed of Aβ peptides and neurofibrillary tangles consisting of the hyperphosphorylated tau protein. The neurodegenerative process in AD is reflected in CSF biomarkers where reduced Aβ levels, along with elevated total tau (t-tau) and phosphorylated tau (p-tau) proteins, signify the ongoing neurodegenerative events. An increase in t-tau levels corresponds to a more rapid onset of cognitive decline, and higher t-tau levels align with higher cognitive decline severity. These findings underscore the significance of tau protein metabolism as a crucial marker in AD’s neurodegenerative cascade. As a result, there is a reconsideration of the amyloid hypothesis cascade, with Aβ still being central in AD pathophysiology. Aβ is currently regarded as an accelerator/initiator, while tau functions are regarded as an executor of the pathogenic process, thereby emphasizing the crucial interaction between them in triggering AD. Recent studies have presented evidence suggesting that cognitive decline and neurodegeneration in cognitively normal individuals are not directly associated with the burden of Aβ levels in CSF. In light of these findings, questions arise regarding the true significance of low CSF Aβ levels in an individual, especially considering the challenge of establishing a direct link between Aβ and neurodegeneration. Additionally, it becomes intriguing to explore the nature of the relationship between Aβ levels and age, prompting further investigation into this aspect [244]. As we delve into the realm of aging and its intricate connection with AD, a profound understanding emerges. Aging, an inevitable biological progression, instigates a cascade of changes impacting cellular function and precipitating physiological shifts. Structural alterations, cardiovascular decline, and heightened intestinal permeability become prominent facets, rendering the elderly more susceptible to infections, cognitive disorders, and gastrointestinal issues. In this complex interplay between healthy aging, inflammation, and the gut microbiota, inflammatory cytokines rise to detrimental levels, fostering chronic illness, disrupting the microbiome, and contributing to cognitive disorders. Embracing the quest for healthy aging, anti-aging foods and supplements, notably probiotics, gain prominence. Probiotics, which are revered for their role in promoting gut health and fortifying the immune system, emerge as pivotal players in the aging narrative. Studies underscore the significance of probiotics, particularly lactic acid bacteria that are sourced from fermented foods, in rectifying age-related imbalances in the gut microbiota. Probiotics showcase their prowess in modulating immune system genes, orchestrating cell differentiation, and unveiling anti-aging effects [245]. Complementing this narrative, vitamins unfold as essential contributors to healthy aging. Vitamin E takes the stage, wielding its prowess in eliminating lipid peroxyl radicals during membrane lipid peroxidation. As a lipophilic radical scavenger, it assumes a crucial role with profound implications for the aging process [246]. Vitamin D, on the other hand, emerges as a key player in brain development and cognitive performance [247]. Proposing a holistic approach, the role of probiotics and vitamins in healthy aging takes center stage, offering a promising avenue to delay the aging process. AD, intrinsically linked to age and predominantly affecting individuals in their 40s to 60s, becomes a focal point. Several probiotic human studies were conducted in the older adult population [120,248,249,250]. Similarly, a number of clinical trials investigating the effects of vitamins were carried out on elderly adults [219,221,251]. As our paper is more focused on preventing and delaying Alzheimer’s disease, we are targeting the administration of probiotics and vitamins as a beneficial regimen for young adult males and females, commencing from the age of 40 and onward. The combination of probiotics and vitamins emerges as a formidable duo that is capable of extending the aging trajectory and indirectly delaying the onset of Alzheimer’s disease. The intricate connection between the roles of probiotics and vitamins in aging and Alzheimer’s disease unveils a synergistic interplay, thereby advocating for a comprehensive approach to holistic wellbeing.

Having said that, it is imperative to acknowledge the limitations and gaps in current research. Furthermore, the complex interplay between gut microbiota, nutrients, and neurological outcomes necessitates a more nuanced understanding through well-designed, longitudinal studies.

In conclusion, the exploration of probiotic and vitamin co-supplementation as a potential strategy for preventing and delaying Alzheimer’s disease marks an exciting frontier in neurotherapeutics. The observed synergistic effects and their broader health implications warrant continued research efforts to elucidate the underlying mechanisms and establish the efficacy of this combinational therapy. As we navigate this evolving landscape, a comprehensive understanding of the interconnections between gut health, nutrition, and neurological wellbeing will be pivotal for advancing therapeutic strategies in the realm of neurodegenerative diseases.

## Abbreviations

1,25(OH)2D	1,25-dihydroxycholecalciferol
25(OH)D	25-hydroxyvitamin D
5HT	5-hydroxytryptamine-Serotonin
AD	Alzheimer’s disease
ALS	Amyotrophic lateral sclerosis
ANS	Autonomic nervous system
ApoE4	apolipoprotein E4
APP	Amyloid precursor protein
Aβ	Amyloid-beta
BACE1	Beta-site APP cleaving enzyme 1
BBB	Blood–brain barrier
BDNF	Brain-derived neurotrophic factor
CAT	Catalase
CD14	Clusters of differentiation Differentiation 14
CFS	Cell-free supernatant
CNS	Central nervous system
CREB	Element-binding protein
CRF	Corticotropin releasing factor
CRH	Corticotropin-releasing hormone
CSF	Cerebrospinal fluid
CTFs	C-terminal fragments
DA	Dopamine
DHA	Docosahexaenoic acid
EECs	Enteroendocrine cells
ENS	Enteric nervous system
EPA	Eicosapentaenoic acid
FAO	Food and Agriculture Organization
GABA	γ-aminobutyric acid
GDNF	Glial cell line-derived neurotrophic factor
GF	Germ-free
GIT	Gastrointestinal tract
GLUT1	Glucose transporter 1
GLUT3	Glucose transporter 3
GPx	Glutathione peroxidase
GSK-3β	Glycogen synthase kinase-3β
GST	Glutathione-S-transferase
HIF-1α	Hypoxia-inducible factor-1α
hMSCs	Mesenchymal stromal cells
HPA	Hypothalamic pituitary adrenal
hs-CRP	High-sensitivity C-reactive protein
IFN-γ	Interferon-gamma
IGF-IRβ	Insulin-like growth factor-I receptor
IL-1	Interleukin-1
IL-6	Interleukin-6
LAB	Lactic acid bacteria
LP	*Lactobacillus plantarum*
LPS	Lipopolysaccharides
MAPT	Gene microtubule-associated protein tau gene
MCI	Mild cognitive impairment
MD-2	Myeloid differentiation factor 2
MDA	Malondialdehyde
MS	Methionine synthase
MS	Multiple sclerosis
MTHFR	Methylenetetrahydrofolate reductase
mTOR	Mammalian target of rapamycin
NADH	Nicotinamide adenine dinucleotide hydrogen
NADPH	Nicotinamide adenine dinucleotide phosphate hydrogen
NFT	Neuronal fibrillary Tangles
NGF	Nerve growth factor
NMDA	*N*-methyl-d-aspartate
NT3	Neurotrophin 3
OGG1	8-Oxoguanine glycosylase
PARP	Poly-ADP ribose polymerase
PCOS	Polycystic Ovary Syndrome
PD	Parkinson’s disease
PHFs	Paired helical filaments
PP2A	Protein phosphatase 2A
PSEN1	Presenilin 1
PSEN2	Presenilin 2
PYY	Peptide YY
RAGE	Receptor for advanced glycation end products
ROS	Reactive oxygen species
RyR	Ryanodine receptor
sAPP	Soluble ectodomain of APP
SCFAs	Short-chain fatty acids
SFs	Straight filaments
SIgA	Secretory IgA
SOD	Superoxide dismutase
SPF	Specific pathogen-free
TAC	Total antioxidant capacity
TLR4	Toll-like receptor 4
TNF-α	Tumor necrosis factor-α
TTP	Tocopherol transfer protein
VDR	Vitamin D receptor
VDRE	Vitamin D receptor element
WHO	The World Health Organization
